# Gradient Learning Algorithms for Ontology Computing

**DOI:** 10.1155/2014/438291

**Published:** 2014-10-29

**Authors:** Wei Gao, Linli Zhu

**Affiliations:** ^1^School of Information and Technology, Yunnan Normal University, Kunming 650500, China; ^2^School of Computer Engineering, Jiangsu University of Technology, Changzhou 213001, China

## Abstract

The gradient learning model has been raising great attention in view of its promising perspectives for applications in statistics, data dimensionality reducing, and other specific fields. In this paper, we raise a new gradient learning model for ontology similarity measuring and ontology mapping in multidividing setting. The sample error in this setting is given by virtue of the hypothesis space and the trick of ontology dividing operator. Finally, two experiments presented on plant and humanoid robotics field verify the efficiency of the new computation model for ontology similarity measure and ontology mapping applications in multidividing setting.

## 1. Introduction and Motivations

The term “ontology” is originally from the field of philosophy and it is used to describe the nature connection of things and the inherent hidden connections of their components. In information and computer science, ontology is a model for knowledge storing and representation and has been widely applied in knowledge management, machine learning, information systems, image retrieval, information retrieval search extension, collaboration, and intelligent information integration. In the past decade, as an effective concept semantic model and a powerful analysis tool, ontology has been widely applied in pharmacology science, biology science, medical science, geographic information system, and social sciences (e.g., see Hu et al., [1], Lambrix and Edberg [2], Mork and Bernstein [3], Fonseca et al., [4], and Bouzeghoub and Elbyed [5]).

The structure of ontology can be expressed as a simple graph. Each concept, object, or element in ontology corresponds to a vertex and each (directed or undirected) edge on an ontology graph represents a relationship (or potential link) between two concepts (objects or elements). Let *O* be an ontology and *G* a simple graph corresponding to *G*. The nature of ontology engineer application can be attributed to get the similarity calculating function which is to compute the similarities between ontology vertices. These similarities represent the intrinsic link between vertices in ontology graph. The goal of ontology mapping is to get the ontology similarity measuring function by measuring the similarity between vertices from different ontologies, such mapping is a bridge between different ontologies, and get a potential association between the objects or elements from different ontologies. Specifically, the ontology similarity function Sim : *V* × *V* → *R* ^+^ ∪ {0} is a semipositive score function which maps each pair of vertices to a nonnegative real number.

**Example 1 .**

Ontology technologies are widely used in humanoid robotics in recent years. Different bionic robot has a different structure. Each bionic robot or each component of a bionic robot can be represented as an ontology. Each vertex in ontology stands for a part or a construction, edge between vertices represents a direct physical link between these constructs, or these parts have intrinsic link with its function. Thus, the similarity calculation between vertices in the same ontology allows us to find the degree of association and the potential link between different constructs in bionic robots. Similarity calculation between two different ontologies (i.e., ontology mapping building) allows us to understand the potential association for different components or parts in two biomimetic robots.

**Example 2 .**

In information retrieval, ontology concepts are often used in query expansion. The user queries the information related concept *A*. If we manually set the parameters *M* > 0, the ontology algorithm will find that all concepts *B* meet Sim(*A*, *B*) > *M*. Then the information related concepts *B* will be returned to the user as the query expansion for concept *A*.

Very recently, ontology technologies are employed in a variety of applications. Ma et al. [6] presented a graph derivation representation based technology for stable semantic measurement. Li et al. [7] raised an ontology representation method for online shopping customers knowledge in enterprise information. Santodomingo et al. [8] proposed an innovative ontology matching system that finds complex correspondences by processing expert knowledge from external domain ontologies and in terms of using novel matching tricks. Pizzuti et al. [9] described the main features of the food ontology and some examples of application for traceability purposes. Lasierra et al. [10] argued that ontologies can be used in designing an architecture for monitoring patients at home.

Traditional methods for ontology similarity computation are heuristic and based on pairwise similarity calculation. With high computational complexity and low intuitive, this model requires large parameters selection. One example of traditional ontology similarity computation method is

(1) SimA,B=α1SimnameA,B+α2SiminstanceA,B+α3Simattribute(A,B)+α4Simstructure(A,B),

where *A* and *B* are two vertices corresponding to two concepts; 0 ≤ *α*_1_, *α*_2_, *α*_3_, *α*_4_ ≤ 1 and ∑_*i*=1_^4^ *α*_*i*_ = 1; Sim_name_, Sim_instance_, Sim_attribute_, and Sim_structure_ are functions of name similarity, instance similarity, attribute similarity, and structure similarity, respectively. These similarity functions are determined by experts directly in terms of their experience. Hence, this model has the following deficiencies:

- many parameters rely heavily on the experts;
- high computational complexity and thus being inapplicable to ontology with large number of vertices;
- pairwise similarities fall reflect the ontology structure intuitively.

Thus, a more advanced way to deal with the ontology similarity computation is using ontology learning algorithm which gets an ontology function *f* : *V* → *R*. By virtue of the ontology function, the ontology graph is mapped into a line which consists of real numbers. The similarity between two concepts then can be measured by comparing the difference between their corresponding real numbers.

The essence of this algorithm is dimensionality reduction. In order to associate the ontology function with ontology application, for vertex *v*, we use a vector to express all its information (including its name, instance, attribute and structure, and semantic information of the concept which is corresponding to the vertex and that is contained in name and attribute components of its vector). In order to facilitate the representation, we slightly confuse the notations and use *v* to denote both the ontology vertex and its corresponding vector. The vector is mapped to a real number by ontology function *f* : *V* → *R*, and the ontology function is a dimensionality reduction operator which maps multidimensional vectors into one-dimensional vectors.

There are several effective methods for getting efficient ontology similarity measure or ontology mapping algorithm in terms of ontology function. Wang et al. [11] considered the ontology similarity calculation in terms of ranking learning technology. Huang et al. [12] raised the fast ontology algorithm in order to cut the time complexity for ontology application. Gao and Liang [13] presented an ontology optimizing model such that the ontology function is determined by virtue of NDCG measure, and it is successfully applied in physics education. Since the large part of ontology structure is the tree, Lan et al. [14] explored the learning theory approach for ontology similarity calculating and ontology mapping in specific setting when the structure of ontology graph has no cycle. In the multidividing ontology setting, all vertices in ontology graph or multiontology graph are divided into *k* parts corresponding to the *k* classes of rates. The rate values of all classes are determined by experts. In this way, a vertex in a rate *a* has larger score than any vertex in rate *b* (if 1 ≤ *a* < *b* ≤ *k*) under the multidividing ontology function *f* : *V* → *R*. Finally, the similarity between two ontology vertices corresponding to two concepts (or elements) is judged by the difference of two real numbers which they correspond to. Hence, the multidividing ontology setting is suitable to get a score ontology function for an ontology application if the ontology is drawn into a noncycle structure. Gao and Xu [15] studied the uniform stability of multidividing ontology algorithm and obtained the generalization bounds for stable multidividing ontology algorithms.

In the above described ontology learning algorithms, their optimal ontology function calculation model or its solution strategy is done by gradient calculation. Specifically, the ontology gradient learning algorithm obtains the ontology function vector $\vec{f}={\left(f^{1},f^{2},\ldots ,f^{n}\right)}^{T}$ which maps each vertex into a real number (the value *f* ^*i*^ corresponds to vertex *v*_*i*_). In this sense, it is good or bad policy gradient calculation algorithm that will determine the merits of the ontology algorithm. In this paper, we raise an ontology gradient learning algorithm for ontology similarity measuring and ontology mapping in multidividing setting. The organization of the rest paper is as follows: the notations and ontology gradient computing model are directly presented in Section 2; the detailed description of new ontology algorithms is shown in Section 3; in Section 4, we obtain some theoretical results concerning the sample error and convergence rate; in Section 5, two simulation experiments on plant science and humanoid robotics are designed to test the efficiency of our gradient computation based ontology algorithm, and the data results reveal that our algorithm has high precision ratio for plant and humanoid robotics applications.

## 2. The Gradient Computation Model for Ontology in Multidividing Setting

In order to combine the machine learning technology and ontology frame, the relevant information for each vertex in ontology graph is represented as an *n*-dimensional vector. Hence the vertex set *V* is a subset of *R* ^*n*^ (vertex space or input space for ontology). Assume that *V* is compact. In the supervised learning, let *Y* = *R* be the label set for *V*. Denote *ρ* as a probability measure on *Z* = *V* × *Y*. Let *ρ*_*V*_ and *ρ*(·∣*v*) be the marginal distribution on *V* and conditional distribution at *v* ∈ *V*, respectively. The ontology function *f*_*ρ*_ : *V* → *R* associated with *ρ* is described as *f*_*ρ*_ = ∫_*Y*_ *ydρ*(*y*∣*v*).

For each vertex *v* ∈ *V*, denote *v* = (*v* ^1^, *v* ^2^,…, *v* ^*n*^)^*T*^ ∈ *R* ^*n*^. Then, the gradient of the ontology function *f*_*ρ*_ is the vector of ontology functions

(2) $$\begin{array}{c} \nabla f_{\rho }={\left(\frac{\partial f_{\rho }}{f_{\rho }v^{1}},\frac{\partial f_{\rho }}{f_{\rho }v^{2}},\ldots ,\frac{\partial f_{\rho }}{f_{\rho }v^{m}}\right)}^{T}. \end{array}$$

Let **z** = {(*v*_*i*_, *y*_*i*_)}_*i*=1_^*m*^ be a random sample independently drawn according to *ρ* in standard ontology setting. The purpose of standard ontology gradient learning is to learn ∇*f*_*ρ*_ from the sample set **z**. From the perspective of statistical learning theory, the gradient learning algorithm is based on the Taylor expansion *f*_*ρ*_(*v*) ≈ *f*_*ρ*_(*v*′)+∇*f*_*ρ*_(*v*′)(*v* − *v*′) if two vertices have large common information (i.e., *v* ≈ *v*′). We expect that *y*_*i*_ ≈ *f*_*ρ*_(*v*) and *y*_*j*_ ≈ *f*_*ρ*_(*u*) if *v*′ = *v*_*i*_′, *v* = *v*_*j*_. The demand *v*_*i*_ ≈ *v*_*j*_ is met by virtue of setting weights

(3) $$\begin{array}{c} w\left(v\right)=w^{s}\left(v\right)=\frac{1}{s^{n+2}}e^{-{\left|v\right|}^{2}/2v^{2}},\\ w_{i,j}=w_{i,j}^{s}=\frac{1}{s^{n+2}}e^{-{\left|v_{i}-v_{j}\right|}^{2}/2v^{2}}=w\left(v_{i}-v_{j}\right). \end{array}$$

Using unknown ontology function vector $\vec{f}={\left(f^{1},f^{2},\ldots ,f^{n}\right)}^{T}$ to replace ∇*f*_*ρ*_, then the standard least-square ontology learning algorithm is denoted as

(4) f→z,λ=argmin⁡f→∈HKn1m2∑i,j=1nwi,jsyi−yj+f→vivj−vi2     +λf→HKn2,

where *λ* and *s* are two positive constants to control the smoothness of ontology function. Here *K* : *V* × *V* → *R* is a positive semidefinite, continuous, and symmetric kernel (i.e., Mercer kernel) and *H*_*K*_ is the reproducing kernel Hilbert space (for short, RKHS) associated with the Mercer kernel *K*. The notation *H*_*K*_^*n*^ presented in (4) is the *n*-fold hypothesis space of *H*_*K*_ composing of vectors of ontology functions $\vec{f}={\left(f^{1},f^{2},\ldots ,f^{n}\right)}^{T}$ with norm ${||\vec{f}||}_{H_{K}^{n}}^{2}={\left\{\sum_{l=1}^{n}{||f^{l}||}_{K}^{2}\right\}}^{1/2}$.

By the representation theory in statistical learning theory, the ontology algorithm (4) can be implemented in terms of solving a linear system for the coefficients {*c*_*i*_, **z**}_*i*=1_^*m*^ of ${\vec{f}}_{z,\lambda }=\sum_{i=1}^{m}c_{i,z}K_{v_{i}}$, where *K*_*v*_(*v*′) = *K*(*v*, *v*′) for *v* ∈ *V* is the ontology function in *H*_*K*_ and *c*_*i*,**z**_ ∈ *R* ^*n*^. Let *d* be the rank of the matrix [*v*_*i*_ − *v*_*m*_]_*i*=1_^*m*−1^; hence the coefficient matrix for the linear system has size *md*. Therefore, this size will become huge if the size of sample set *m* is large itself. The standard approximation ontology algorithm allows us to solve linear systems with coefficient matrices of smaller sizes.

The gradient learning model for ontology algorithm in standard setting is determined as follows:

(5) f→t+1z=f→tz−ηtm2∑i,j=1mwi,jsyi−yj+f→tzvi·vj−viKvi−ηtλtf→tz,

where the sample set **z** ∈ *Z* ^*m*^, ${\vec{f}}_{1}^{z}=0$, *t* ∈ *Z*, {*η*_*t*_} is the sequence of step sizes and {*λ*_*t*_} is the sequence of balance parameters.

For multidividing ontology setting, the vertex in ontology sample set can be divided into *k* rates. Let **z** = {**z** ^1^, **z** ^2^,…, **z** ^*k*^} with **z** ^*i*^ = {*v*_1_^*i*^, *v*_2_^*i*^,…, *v*_*m*_*k*__^*i*^} for 1 ≤ *i* ≤ *k*. Denote |**z**_*a*_ | = *m*_*a*_, *m* = ∑_*i*=1_^*k*^ *m*_*i*_ and *y*_*i*_^*a*^ is the label of *v*_*i*_^*a*^ for 1 ≤ *a* ≤ *k* and 1 ≤ *i* ≤ *m*_*a*_. Hence, (4) becomes

(6) f→z,λ=argmin⁡f→∈HKnηt∑a=1k−1∑b=i+1kmamb     ×∑a=1k−1 ∑b=i+1k‍ ∑i=1ma ‍∑j=1mbw  ia,jbs     ×yia−yjb+f→viavjb−via2+λf→HKn2.

We obtain the following gradient computation model for ontology application in multidividing setting which corresponds to (5):

(7) f→t+1z=f→tz−ηt∑a=1k−1∑b=i+1kmamb×∑a=1k−1 ‍∑b=i+1k ‍∑i=1ma ‍∑j=1mbw  ia,jbs×yia−yjb+f→tzvia·vjb−viaKvia−ηtλtf→tz.

Here in (6) and (7), *w*__*i*_^*a*^,_*j*_^*b*^_^(*s*)^ = (1/*s* ^*n*+2^)*e* ^−((*v*_*i*_^*a*^)^2^ − (*v*_*j*_^*b*^)^2^)/2*s*^2^^.

We emphasize that our algorithm in multidividing setting is different from that of Wu et al. [16]. First, the label *y* for ontology vertex *v* is used to present its class information in [16], that is, *y* ∈ {1,…, *k*}, while in our setting, *y* ∈ *R*. Second, the computation model in [16] relies heavily on the convexity loss function *l*, while our algorithm depends on the weight function *w*.

## 3. Description of Ontology Algorithms via Gradient Learning

The above raised gradient learning ontology algorithm can be used in ontology concepts similarity measurement and ontology mapping. The basic idea is the following: via the ontology gradient computation model, the ontology graph is mapped into a real line consisting of real numbers. The similarity between two concepts then can be measured by comparing the difference between their corresponding real numbers.

**Algorithm 3 (gradient calculating based ontology similarity measure algorithm).**

For *v* ∈ *V*(*G*) and *f* is an optimal ontology function determined by gradient calculating, we use one of the following methods to obtain the similar vertices and return the outcome to the users.

*Method 1.* Choose a parameter *U* and return set {*v*′ ∈ *V*(*G*), |*f*(*v*′) − *f*(*v*)|≤*U*}.

*Method 2.* Choose an integer *U* and return the closest *N* concepts on the value list in *V*(*G*).

Clearly, method 1 looks like fairer, but method 2 can control the number of vertices that return to the users.

**Algorithm 4 (gradient calculating based ontology mapping algorithm).**

Let *G*_1_, *G*_2_,…, *G*_*d*_ be ontology graphs corresponding to ontologies *O*_1_, *O*_2_,…, *O*_*d*_. For *v* ∈ *V*(*G*_*i*_) (1 ≤ *i* ≤ *d*) and *f* being an optimal ontology function determined by gradient calculating, we use one of the following methods to obtain the similar vertices and return the outcome to the users.

*Method 1.* Choose a parameter *U* and return set {*v*′ ∈ *V*(*G* − *G*_*i*_), |*f*(*v*′) − *f*(*v*)|≤*U*}.

*Method 2.* Choose an integer *N* and return the closest *N* concepts on the list in *V*(*G* − *G*_*i*_).

Also, method 1 looks like fairer and method 2 can control the number of vertices that return to the users.

## 4. Theoretical Analysis

In this section, we give certain theoretical analysis for our proposed multidividing ontology algorithm. Let $\kappa =\sup_{v\in V}\sqrt{K(v,v)}$ and Diam(*V*) = sup⁡_*v*,*v*′∈*V*_ | *v* − *v*′|. We divide this section into two parts: first, some useful lemmas are prepared; then, main results in our paper concerning approximation conclusions are presented. Our error analysis depends on integral operators and gradient learning, and more references on these tricks can be referred to in Mukherjee and Wu [18], Mukherjee et al. [19], Yao et al. [20], and Rosasco et al. [21].

Set

(8) f→λ∗=argmin⁡f→∈HKn∑a=1k−1 ‍∑b=a+1k∫Za∫Zbw(va−vb)            ×ya−yb+f→v              ·vb−va22dρ            ×(va,ya)dρ(vb,yb)            +λf→HKn2.

In what follows, *m*_Π_ = *m*_1_ *m*_2_ ⋯ *m*_*k*_,

(9) mΠ1=mΠm1=m2m3⋯mk,mΠ2=mΠm2=m1m3⋯mk, ⋮,mΠk=mΠmk=m1m2⋯mk−1.

Our tricks of proofs in this paper follow from [22, 23].

### 4.1. Preliminary Results

Let sequence ${\left\{{\vec{f}}_{t}\right\}}_{t\in N}$ be the noise-free limit of the sequence (7) which is determined by ${\vec{f}}_{1}=0$ and

(10) f→t+1=f→t−ηt∑a=1k−1‍ ∑b=a+1k∫Za∫Zbw(va−vv)         ×ya−yb+f→tv           ·vb−va         ×vb−vaKvdρva,yadρvb,yb         −ηtλtf→t.

Our error analysis for proving main result (Theorems 12 and 13 in the next subsection) consists of two parts: sample error and approximation error.

The main task in this subsection is to estimate the sample error $||{\vec{f}}_{t}^{z}-{\vec{f}}_{t}||$ in terms of McDiarmid-Bernstein-type probability inequality and the multidividing sampling operator. For each 1 ≤ *a* ≤ *k*, the multidividing sampling operator *S*_*v*_^*a*^ : *H*_*K*_^*n*^ → *R* ^*m*_*a*_*n*^ associated with a discrete subset **v** ^*a*^ = {*v*_*i*_^*a*^}_*i*=1_^*m*_*a*_^ of *V* is defined by

(11) $$\begin{array}{c} S_{v}^{a}\left(\vec{f}\right)={\left(\vec{f}\left(v_{i}^{a}\right)\right)}_{i=1}^{m_{a}}={\left(\vec{f}\left(v_{1}^{a}\right),\vec{f}\left(v_{2}^{a}\right),\ldots ,\vec{f}\left(v_{m_{a}^{a}}\right)\right)}^{T}. \end{array}$$

The adjoint of the multidividing ontology sampling operator, (*S*_**v**_^*a*^)^*T*^ : *R* ^*m*_*a*_*n*^ → *H*_*K*_^*n*^, is given by

(12) $$\begin{array}{c} {\left(S_{v}^{a}\right)}^{T}\left(c\right)=\overset{m_{a}}{\underset{i=1}{\sum }}c_{i}^{a}K_{v_{i}^{a}}, \end{array}$$

where

(13) $$\begin{array}{c} c={\left(c_{i}\right)}_{i=1}^{m_{a}}={\left(c_{1},c_{2},\ldots ,c_{m_{a}}\right)}^{T}\in R^{m_{a}n}. \end{array}$$

Let us express (7) by virtue of the multidividing ontology sampling operator. Note that

(14) f→tzvia·vjb−viavjb−via  =(vjb−via)vjb−viaTf→tz(via)  =(vjb−via)vjb−viaTSvf→tzia.

For each pair of (*a*, *b*) with 1 ≤ *a* < *b* ≤ *k*, we single out one summation ∑_*j*=1_^*m*_*b*_^ from (7) as

(15) $$\begin{array}{c} B_{i}^{a,b}=\overset{m_{b}}{\underset{j=1}{\sum }}w_{{\;}_{i}^{a}{,}_{j}^{b}}\left(v_{j}^{b}-v_{i}^{a}\right){\left(v_{j}^{b}-v_{i}^{a}\right)}^{T}\in R^{n\times n},\\ Y_{i}^{a,b}=\overset{m_{b}}{\underset{j=1}{\sum }}w_{{\;}_{i}^{a}{,}_{j}^{b}}\left(y_{j}^{b}-y_{i}^{a}\right){\left(v_{j}^{b}-v_{i}^{a}\right)}^{T}\in R^{n}. \end{array}$$

We infer that

(16) f→t+1z=1−ηtλtf→tz−ηt∑a=1k−1∑b=a+1kmamb×−∑i=1ma ‍∑j=1mbYia,bKvia+∑i=1ma ∑j=1mbKviaBia,bf→tzvia.

Denote

(17) $$\begin{array}{c} {\left(D_{v}^{a}\right)}^{a,b}=\mathrm{diag}\left\{B_{1}^{a,b},B_{2}^{a,b},\ldots ,B_{m_{a}}^{a,b}\right\}\in R^{m_{a}n\times m_{a}n}\\ {\left({\vec{Y}}^{a}\right)}^{a,b}={\left(Y_{1}^{a,b},Y_{2}^{a,b},\ldots ,Y_{m_{a}}^{a,b}\right)}^{T}\in R^{m_{a}n}. \end{array}$$

Hence, we have

(18) f→t+1z=1−ηtλtf→tz+ηt∑a=1k−1∑b=a+1kmamb×∑a=1k−1 ∑b=a+1kSvaTY→aa,bT−ηt∑a=1k−1∑b=a+1kmamb×∑a=1k−1 ∑b=a+1kSvaTDvaa,bSvaf→tz.

Thus, it confirms the following representation for the sequence $\left\{{\vec{f}}_{t}^{z}\right\}$. For simplicity, let ∏_*q*=*t*+1_^*t*^(*I* − *L*_**v**,*q*_) = *I* in the following contents.

**Lemma 5 .**

Set

(19) Lv,t=ηt∑a=1k−1∑b=a+1kmamb∑a=1k−1 ∑b=a+1kSvaTDvaa,bSva+ηtλtI.

If $\left\{{\vec{f}}_{t}^{z}\right\}$ is defined by (7), we deduce

(20) f→tz=Πi=1t−1I−Lv,if→1z+∑i=1t−1 ∏q=i+1t−1I−Lv,qηt∑a=1k−1∑b=a+1kmamb×∑a=1k−1 ∑b=a+1kSvaTY→aa,bT.

We should discuss the convergence of the multidividing ontology operator

(21) $$\begin{array}{c} \frac{1}{\sum_{a=1}^{k-1}\sum_{b=a+1}^{k}m_{a}m_{b}}\left\{\overset{k-1}{\underset{a=1}{\sum }}\;\overset{k}{\underset{b=a+1}{\sum }}{\left(S_{v}^{a}\right)}^{T}{\left(D_{v}^{a}\right)}^{a,b}S_{v}^{a}\left({\vec{f}}_{t}^{z}\right)\right\} \end{array}$$

to the integral operator *L*_*K*,*s*_ : *H*_*K*_^*n*^ → *H*_*K*_^*n*^ determined by

(22) LK,sf→=∑a=1k−1 ∑b=a+1k∫Va∫Vbw(va−vb)(vb−va)vb−vaT       ·f→vaKvadρVavadρVbvb,

where $\vec{f}\in H_{K}^{n}$.

**Lemma 6 .**

Let **z** = {**z**_1_, **z**_2_,…, **z**_*k*_} be multidividing sample set independently drawn according to a probability distribution *ρ* on *Z*. Denote (*H*, ||·||) as a Hilbert space and suppose that *F* : *Z* ^*m*_1_×*m*_2_×⋯×*m*_*k*_^ → *H* is measurable. If there is nonnegative $\tilde{M}$ such that $||F(z)-Ez(F(v))||\leq \tilde{M}$ for each *v* ∈ **z** and almost every **z** ∈ *Z* ^*m*_1_×*m*_2_×⋯×*m*_*k*_^, then for every *ɛ* > 0,

(23) Pz∈Zm1×m2×⋯×mkFz−EzFz≥ɛ  ≤2exp⁡{−ɛ22(M~ɛ+σ2)},

where

(24) $$\begin{array}{c} \sigma^{2}=\overset{k}{\underset{a=1}{\sum }}\;\overset{m_{a}}{\underset{i=1}{\sum }}\underset{z\setminus \{v_{i}^{a}\}\in Z^{m_{1}\times m_{2}\times \cdots \times (m_{a}-1)\times \cdots \times m_{k}}}{\sup}E_{v_{i}}\left\{{||F(z)-E_{v_{i}}(F(z))||}^{2}\right\}. \end{array}$$

For any 0 < *δ* < 1, with confidence 1 − *δ*, one gets

(25) Fz−EzFz≤4log⁡4δM~+σ2≤4(1+mΠ∑i=1kmΠi)log⁡4δM~.

By regarding $\left(1/\sum_{a=1}^{k-1}\sum_{b=a+1}^{k}m_{a}m_{b}\right)\{\sum_{a=1}^{k-1}\sum_{b=a+1}^{k}{\left(S_{v}^{a}\right)}^{T}{\left(D_{v}^{a}\right)}^{a,b}S_{v}^{a}({\vec{f}}_{t}^{z})\}$ and *L*_*K*,*s*_ as elements in (*L*(*H*_*K*_^*n*^) and ||·||_*L*(*H*_*K*_^*n*^)_, the space of bounded linear multidividing ontology operators on *H*_*K*_^*n*^, Lemma 6 cannot be directly employed because *L*(*H*_*K*_^*n*^) is not a Hilbert space, but a Banach space only. Therefore, we consider a subspace of *L*(*H*_*K*_^*n*^), *HS*(*H*_*K*_^*n*^) which is the space of Hilbert-Schmidt operators on *H*_*K*_^*n*^ with inner product 〈*A*, *B*〉_*HS*(*H*_*K*_^*n*^)_ = *Tr*(*B* ^*T*^ *A*). As *HS*(*H*_*K*_^*n*^) is a subspace of *L*(*H*_*K*_^*n*^), their norm relations are presented as

(26) $$\begin{array}{c} {||A||}_{L(H_{K}^{n})}\leq {||A||}_{HS(H_{K}^{n})},\\ {||AB||}_{HS(H_{K}^{n})}\leq {||A||}_{HS(H_{K}^{n})}{||B||}_{HS(H_{K}^{n})}. \end{array}$$

In addition, *HS*(*H*_*K*_^*n*^) is a Hilbert space and contains multidividing ontology operators *L*_*K*,*s*_ and $\left(1/\sum_{a=1}^{k-1}\sum_{b=a+1}^{k}m_{a}m_{b}\right)\{\sum_{a=1}^{k-1}\sum_{b=a+1}^{k}{\left(S_{v}^{a}\right)}^{T}{\left(D_{v}^{a}\right)}^{a,b}S_{v}^{a}({\vec{f}}_{t}^{z})\}$. By applying Lemma 6 to this Hilbert space, we obtain the following lemma.

**Lemma 7 .**

Let **v** = {**v** ^1^, **v** ^2^,…, **v** ^*k*^} be multidividing sample set independently drawn from (*V*, *ρ*_*V*_). With confidence 1 − *δ*, one obtains

(27) 1∑a=1k−1∑b=a+1kSva,bTDvSva,b−LK,sHSHKn ≤34nκ2
Diam
V2mΠ/∑i=1kmΠisn+2log⁡4δ.

**Proof**

Let *H* = *HS*(*H*_*K*_^*n*^). Consider the multidividing ontology function *F* : *V* ^*m*_1_×*m*_2_×⋯×*m*_*k*_^ → *H* with values in *H* = *HS*(*H*_*K*_^*n*^) defined by

(28) $$\begin{array}{c} F\left(v\right)=\frac{1}{\sum_{a=1}^{k-1}\sum_{b=a+1}^{k}m_{a}m_{b}}\left\{\overset{k-1}{\underset{a=1}{\sum }}\;\overset{k}{\underset{b=a+1}{\sum }}{\left(S_{v}^{a}\right)}^{T}{\left(D_{v}^{a}\right)}^{a,b}S_{v}^{a}\right\}. \end{array}$$

For $\vec{f}\in H_{K}^{n}$, we confirm that

(29) Fvf→=1∑a=1k−1∑b=a+1k∑i=1ma ∑j=1mbwvia−vjbvjb−via        ×vjb−viaTf→(via)Kvia.

Recall that reproducing property of the RKHS *H*_*K*_ says that

(30) $$\begin{array}{c} f\left(v\right)={\langle f,K_{v}\rangle }_{K},\;\forall v\in V,\;\;f\in H_{K}. \end{array}$$

It implies that the rank of operator *A*_*v*_ : *H*_*K*_ → *H*_*K*_ determined by *A*_*v*_(*f*) = *f*(*v*)*K*_*v*_ = 〈*f*, *K*_*v*_〉_*K*_ *K*_*v*_ is 1, and also in *HS*(*H*_*K*_). Furthermore, ||*A*_*v*_||*HS*(*H*_*K*_) = *K*(*v*, *v*). Let ${\vec{A}}_{v}$ be the operator on *H*_*K*_^*n*^ which maps $\vec{f}$ to $\vec{f}(v)K_{v}$. Then the above fact reveals that ${||{\vec{A}}_{v}||}_{HS(H_{K}^{n})}\leq K(v,v)\sqrt{n}$. Hence for any **v** ∈ *V* ^*m*_1_×*m*_2_×⋯×*m*_*k*_^, we infer that

(31) Fv=1∑a=1k−1∑b=a+1k∑i=1ma ‍ ∑j=1mbwvia−vjbvjb−via        ×vjb−viaTA→via∈HS(HKn).

Using the fact that *w*(*v*) ≤ 1/*s* ^*n*+2^ and ${||{\vec{A}}_{v}||}_{HS(H_{K}^{n})}\leq \sqrt{n}K(v,v)\leq \sqrt{n}\kappa^{2}$, we deduce that

(32) Fv−EviFvHSHKn ≤4(mΠ/∑i=1kmΠi−1)κ2DiamV2nmΠ/∑i=1kmΠi2sn+2.

Since

(33) Ev1∑a=1k−1∑b=a+1kmamb∑a=1k−1 ∑b=a+1kSvaTDvaa,bSva=Ev(F(v))=mΠ/∑i=1kmΠimΠ/∑i=1kmΠi−1LK,s,

the stated result is held by combining Lemma 6 with

(34) $$\begin{array}{c} \tilde{M}={\left(\text{Diam}\left(V\right)\right)}^{2}\kappa^{2}\sqrt{n}\frac{8\left(m_{\Pi }/\sum_{i=1}^{k}m_{\Pi }^{i}-1\right)}{{\left(m_{\Pi }/\sum_{i=1}^{k}m_{\Pi }^{i}\right)}^{2}s^{n+2}} \end{array}$$

and using the bound ${||L_{K,s}||}_{HS(H_{K}^{n})}\leq \kappa^{2}\sqrt{n}{\left(\text{Diam}(V)\right)}^{2}/s^{n+2}$.

In order to find the difference between ${\vec{f}}_{t}^{z}$ and ${\vec{f}}_{t}$, the convergence of

(35) $$\begin{array}{c} \frac{1}{\sum_{a=1}^{k-1}\sum_{b=a+1}^{k}m_{a}m_{b}}\left\{\overset{k-1}{\underset{a=1}{\sum }}\;\overset{k}{\underset{b=a+1}{\sum }}{\left(S_{v}^{a}\right)}^{T}{\left({\left({\vec{Y}}^{a}\right)}^{a,b}\right)}^{T}\right\} \end{array}$$

to the ontology function defined by (55) is studied.

**Lemma 8 .**

Let **z** be a multidividing ontology sample independently drawn from (*Z*, *ρ*). With confidence 1 − *δ*, one has

(36) 1∑a=1k−1∑b=a+1kmamb∑a=1k−1 ∑b=a+1kSvaTY→aa,bT−f→ρ,sHKn≤68
Diam
(V)MκmΠ/∑i=1kmΠisn+2log⁡4δ.

**Proof**

By applying Lemma 6 to the Hilbert space *H* = *H*_*K*_^*n*^ and the ontology function *F* : *Z* ^*m*_1_×*m*_2_×⋯×*m*_*k*_^ → *H*_*K*_^*n*^ given by

(37) Fz=1∑a=1k−1∑b=a+1kmamb∑a=1k−1 ∑b=a+1kSvaTY→aa,bT=1∑a=1k−1∑b=a+1k∑i=1ma∑j=1mbwvia−vjbvjb−viavjb−viaKvia,

we yield $E_{z}(F(z))=(\left(m_{\Pi }/\sum_{i=1}^{k}m_{\Pi }^{i}-1\right)/m_{\Pi }/\sum_{i=1}^{k}m_{\Pi }^{i}){\vec{f}}_{\rho ,s}$. Hence, for almost every **z** ∈ *Z* ^*m*_1_×*m*_2_×⋯×*m*_*k*_^, we get

(38) Fz−EziFzHKn ≤16MκDiam(V)(mΠ/∑i=1kmΠi−1)mΠ/∑i=1kmΠi2sn+2.

Lemma 6 implies that for any 0 < *δ* < 1, with confidence 1 − *δ*, we obtain

(39) 1∑a=1k−1∑b=a+1kmamb∑a=1k−1 ∑b=a+1kSvaTY→aa,bT −mΠ/∑i=1kmΠi−1mΠ/∑i=1kmΠif→ρ,sHKn  ≤321+1/mΠ/∑i=1kmΠiMκDiam(V)mΠ/∑i=1kmΠisn+2log⁡4δ.

Finally, conclusion follows from the fact that ${||{\vec{f}}_{\rho ,s}||}_{H_{K}^{n}}\leq 4\text{Diam}(V)M\kappa /s^{n+2}$.

Obviously, for $\left\{{\vec{f}}_{t}^{z}\right\}$, the sequence $\left\{{\vec{f}}_{t}\right\}$ has a similar expression as (20).

**Lemma 9 .**

Let *L*_*K*,*λ*_*i*_,*η*_*i*__ = *η*_*i*_ *L*_*K*,*s*_ + *η*_*i*_ *λ*_*i*_ *I* be an ontology operator on *H*_*K*_^*n*^ and suppose that ∏_*q*=*i*+1_^*t*−1^(*I* − *L*_*K*,*λa*_*k*_,*η*_*k*__) = *I*. For the ontology operator *L*_*K*,*s*_ determined by (22) and $\left\{{\vec{f}}_{t}\right\}$ by (10), one obtains

(40) $$\begin{array}{c} {\vec{f}}_{t}=\overset{t-1}{\underset{i=1}{\prod }}\left(I-L_{K,\lambda_{i},\eta_{i}}\right){\vec{f}}_{1}+\overset{t-1}{\underset{i=1}{\sum }}\;\overset{t-1}{\underset{q=i+1}{\prod }}\left(I-L_{K,\lambda_{k},\eta_{k}}\right)\eta_{i}{\vec{f}}_{\rho ,s}. \end{array}$$

The sample error ${||{\vec{f}}_{t}^{z}-{\vec{f}}_{t}||}_{H_{K}^{n}}$ is stated in the following conclusion.

**Theorem 10 .**

Let $\left\{{\vec{f}}_{t}^{z}\right\}$ be obtained by (5) and $\left\{{\vec{f}}_{t}\right\}$ by (10). Suppose that *η*_*i*_ ≤ 1 and *λ*_*i*+1_ ≤ *λ*_*i*_ ≤ 1 for all *i* ∈ *N*. Then for any 0 < *δ* < 1, with confidence 1 − *δ*, one infers that

(41) f→tz−f→tHKn≤34
Diam
VκmΠ/∑i=1kmΠiλt−12sn+2 ×κn
Diam
V+4λt−1Mlog⁡8δ.

**Proof**

Let

(42) $$\begin{array}{c} {\vec{f}}_{\rho ,t}^{z}=\overset{t-1}{\underset{i=1}{\sum }}\;\overset{t-1}{\underset{q=i+1}{\prod }}\left(I-L_{v,k}\right)\eta_{i}{\vec{f}}_{\rho ,s}+\overset{t-1}{\underset{i=1}{\prod }}\left(I-L_{v,i}\right){\vec{f}}_{1}^{z}. \end{array}$$

Let *Z*_1_⊆*Z* ^*m*_1_×*m*_2_×⋯×*m*_*k*_^ with measure at least 1 − *δ* such that (36) establishes for any **z** ∈ *Z*_1_. Thus, from the positivity of the multidividing ontology operator (*S*_**v**_^*a*^)^*T*^(*D*_**v**_^*a*^)^*a*,*b*^ *S*_**v**_^*a*^ (for each pair of (*a*, *b*)) on *H*_*K*_^*n*^ and the assumption ∏_*q*=*t*+1_^*t*−1^(1 − *η*_*q*_ *λ*_*q*_) = 1, we have that for any **z** ∈ *Z*_1_,

(43) f→tz−f→ρ,tzHKn=∑i=1t−1 ∏q=i+1t−1I−Lv,qηi  ×1∑a=1k−1∑b=a+1kmamb    ×∑a=1k−1 ∑b=a+1kSvaTY→aa,bT−f→ρ,sLHKn ≤∑i=1t−1 ∏q=i+1t−1I−Lv,kLHKn68
Diam
VMκmΠ/∑i=1kmΠisn+2log⁡4δ ≤68
Diam
(V)MκmΠ/∑i=1kmΠisn+2log⁡4δ∑i=1t−1 ‍∏q=i+1t−1(1−ηqλq)ηi.

In terms of *η*_*i*_ *λ*_*i*_ = 1 − (1 − *η*_*i*_ *λ*_*i*_) and 1 ≤ *λ*_*i*_ *λ*_*t*−1_^−1^, we get

(44) ∑i=1t−1 ∏q=i+1t−11−ηqλqηi ≤1λt−1∑i=1t−1 ∏q=i+1t−11−ηqλq−∑i=1t−1 ∏q=it−11−ηqλq =1λt−1{1−∏q=1t−1(1−ηqλq)}.

By virtue of the assumptions on *η*_*i*_, *λ*_*i*_, we infer that

(45) $$\begin{array}{c} \overset{t-1}{\underset{i=1}{\sum }}\;\overset{t-1}{\underset{q=i+1}{\prod }}\left(1-\eta_{q}\lambda_{q}\right)\eta_{i}\leq \frac{1}{\lambda_{t-1}}, \end{array}$$

which implies that

(46) $$\begin{array}{c} {||{\vec{f}}_{t}^{z}-{\vec{f}}_{\rho ,t}^{z}||}_{H_{K}^{n}}\leq \log\frac{4}{\delta }\frac{68\text{ Diam }\left(V\right)M\kappa }{s^{n+2}\sqrt{m_{\Pi }/\sum_{i=1}^{k}m_{\Pi }^{i}}\lambda_{t-1}} \end{array}$$

for any **z** ∈ *Z*_1_.

Now, we consider the estimate of ${||{\vec{f}}_{t}^{z}-{\vec{f}}_{\rho ,t}^{z}||}_{H_{K}^{n}}$. Let *Z*_2_⊆*Z* ^*m*_1_×*m*_2_×⋯×*m*_*k*_^ with measure at least 1 − *δ* such that (27) is established for any **z** ∈ *Z*_2_. In view of (26), for each **z** ∈ *Z*_2_ we yield

(47) 1∑a=1k−1∑b=a+1kmamb∑a=1k−1 ∑b=a+1kSvaTDvaa,bSva−LK,sLHKn ≤log⁡2δ34nκ2
Diam
V2sn+2mΠ/∑i=1kmΠi.

Using the fact that *L*_*K*,*λ*_*j*_,*n*_*j*__ − *L*_**v**,*j*_ = *η*_*j*_(*L*_*K*,*s*_ − (1/∑_*a*=1_^*k*−1^∑_*b*=*a*+1_^*k*^ *m*_*a*_ *m*_*b*_){∑_*a*=1_^*k*−1^∑_*b*=*a*+1_^*k*^(*S*_**v**_^*a*^)^*T*^(*D*_**v**_^*a*^)^*a*,*b*^ *S*_**v**_^*a*^}), we obtain that for any **z** ∈ *Z*_2_,

(48) f→t−f→ρ,tzHKn=∑i=1t−1∏q=i+1t−1I−Lv,q−∏l=i+1t−1I−LK,λl,njηif→ρ,sHKn=∑i=1t−1 ∑j=i+1t−1 ∏q=j+1t−1I−Lv,qLK,λj,nj−Lv,q   ×∏l=i+1t−1I−LK,λl,njηif→ρ,sHKn≤∑i=1t−1 ∑j=i+1t−1 ∏q=j+1t−11−ηqλqηj ×17κ2
Diam
V2nmΠ/∑i=1mi'sn+2log⁡2δ∏l=i+1j−1(1−ηlλl)ηif→ρ,sHKn.

By changing the order of summation, we determine that

(49) f→t−f→ρ,tzHKn ≤34κ2
Diam
V2nmΠ/∑i=1kmΠisn+2log⁡4δ  ·∑j=2t−1 ∏q=j+1t−11−ηqλqηj  ×∑i=1j−1 ∏l=i+1j−1(1−ηlλl)ηif→ρ,sHKn.

According to (45), we can verify that ${||{\vec{f}}_{t}-{\vec{f}}_{\rho ,t}^{z}||}_{H_{K}^{n}}$ is bounded by

(50) log⁡4δ34κ2
Diam
V2nmΠ/∑i=1kmΠisn+2  ×∑j=2t−1 ∏q=j+1t−11−ηqλqηj1λj−1f→ρ,sHKn ≤log⁡(4δ)34κ2
Diam
V2nmΠ/∑i=1kmΠisn+21λj−12f→ρ,sHKn.

In view of the above fact and (46), we obtain that for any **z** ∈ *Z*_1_∩*Z*_2_,

(51) f→t−f→ρ,tzHKn  ≤log⁡2δ68κ2
Diam
V2nmΠ/∑i=1kmΠisn+2      +34κ2
Diam
V2nmΠ/∑i=1kmΠisn+2f→ρ,sHKn.

However, the measure of the subset *Z*_1_∩*Z*_2_ of *Z* ^*m*_1_×*m*_2_×⋯×*m*_*k*_^ is at least 1 − 2*δ*. The desired conclusion follows after substituting *δ* for *δ*/2.

The following result is Theorem 4 in Dong and Zhou [23]; it also holds in multidividing setting and we skip the detailed proof.

**Theorem 11 .**

Let {*λ*_*t*_, *η*_*t*_}_*t*∈*N*_ be determined by (53). Then, we deduce that

(52) f→t−f→λt∗HKn≤t2γ+α−14γCλ1η1,γ+α,1−γ+exp⁡λ1η1−log⁡⁡eλ1η11−γ−α ×f→ρ,sHKnλ1.

### 4.2. Main Results

The first main result in our paper implies that $\left\{{\vec{f}}_{t}^{z}\right\}$ is a good approximation of a noise-free limit for the ontology function (6) as a solution of (8) which we refer as multidividing ontology function ${\vec{f}}_{\lambda }^{*}$.

**Theorem 12 .**

Let 0 < *γ*, *α* < 1, and *λ*_1_ and *η*_1_ > 0 satisfy 2*γ* + *α* < 1 and *λ*_1_ *η*_1_ < 1. For any *t* ∈ *N*, take

(53) $$\begin{array}{c} \lambda_{t}=\lambda_{1}t^{-\alpha }. \end{array}$$

Define $\left\{{\vec{f}}_{t}^{z}\right\}$ by (7) and $\left\{{\vec{f}}_{\lambda }^{*}\right\}$ by (8). If |*y* | ≤*M* is almost established, then for any 0 < *δ* < 1, with confidence 1 − *δ*, one has

(54) f→tz−f→λt∗HKn≤C~log⁡8δt2γmΠ/∑i=1kmΠisn+2+t2γ+α−1×1+f→ρ,sHKn,

where constant $\tilde{C}$ independent of *m*_1_, *m*_2_, …, *m*_*k*_, *t*, *s* or *δ* and ${\vec{f}}_{\rho ,s}$ is the multidividing ontology function determined by

(55) f→ρ,s=∑a=1k−1 ∑b=a+1k∫Va∫Vbwa,bsva−vbfρvb−fρva       ×(vb−va)KvdρV(va)dρV(vb).

The proof of Theorem 12 follows from Theorems 10 and 11 and an exact expression for the constant $\tilde{C}$ relying on *α*, *η*_1_, *λ*_1_, *κ*, *n*, *γ*, *M* and Diam (*V*) can be easily determined.

The second main result in our paper follows from Theorem 10 and the technologies raised in [23].

**Theorem 13 .**

Assume that for certain 0 < *τ* ≤ 2/3, *c*_*ρ*_ > 0 and for any *s* > 0, the marginal distribution *ρ*_*V*_ satisfies

(56) $$\begin{array}{c} \rho_{V}\left(\left\{v\in V:\underset{u\in R^{n}\setminus V}{\inf}\left|u-v\right|\leq s\right\}\right)\leq c_{\rho }^{2}s^{4s}, \end{array}$$

and the density *p*(*v*) of *dρ*_*V*_(*v*) exists and for any, any *u*, *v* ∈ *V* satisfies

(57) $$\begin{array}{c} \underset{v\in V}{\sup}p\left(v\right)\leq c_{\rho },\;\;\left|p\left(v\right)-p\left(u\right)\right|\leq c_{\rho }{\left|u-v\right|}^{\tau }. \end{array}$$

Suppose that the kernel *K* ∈ *C* ^3^ and ∇*f*_*ρ*_ ∈ *H*_*K*_^*n*^. Let 0 < *β* < 1/(4 + (2*n* + 4)*γ*/*τ*) and 0 < *γ* < 2/5. Take *λ*_*t*_ = *t* ^−*γ*^, *η*_*t*_ = *t* ^(5/2)*γ*−1^, and *s* = *s*(*m*_1_, *m*_2_,…, *m*_*k*_) = (*κc*_*ρ*_)^2/*τ*^(*m*_Π_/∑_*i*=1_^*k*^ *m*_Π_^*i*^)^−*βγ*/*τ*^ and suppose that (*m*_Π_/∑_*i*=1_^*k*^ *m*_Π_^*i*^)^*β*^ ≤ *t* ≤ 2(*m*_Π_/∑_*i*=1_^*k*^ *m*_Π_^*i*^)^*β*^; then for any 0 < *δ* < 1, with confidence 1 − *δ*, one infers that

(58) $$\begin{array}{c} {||{\vec{f}}_{t}^{z}-\nabla f_{\rho }||}_{{\left(L_{\rho_{V}^{2}}\right)}^{n}}\leq {\tilde{C}}_{\rho ,K}{\left(\frac{1}{m_{\Pi }/\sum_{i=1}^{k}m_{\Pi }^{i}}\right)}^{\theta }\log\left(\frac{4}{\delta }\right), \end{array}$$

where

(59) $$\begin{array}{c} \theta =\min\left\{\frac{1}{2}-2\beta -\frac{\left(n+2\right)\beta \gamma }{\tau },\frac{\beta \gamma }{2}\right\} \end{array}$$

and constant ${\tilde{C}}_{\rho ,K}$ is independent of *m*_1_, *m*_2_, …, *m*_*k*_, *t* or *δ*.

**Proof**

Obviously, under the assumptions *K* ∈ *C* ^3^, (56) and (57), we get

(60) $$\begin{array}{c} {||{\vec{f}}_{\rho ,s}||}_{H_{K}^{n}}\leq C_{\rho ,K}\left(c_{\rho }n{\left(2\pi \right)}^{n/2}\kappa^{2}{||\nabla f_{\rho }||}_{H_{K}^{n}}+s\right). \end{array}$$

Furthermore, by virtue of Proposition 15 in Mukherjee and Zhou [22], we have

(61) $$\begin{array}{c} {||{\vec{f}}_{t}^{*}-\nabla f_{\rho }||}_{{\left(L_{\rho_{V}^{2}}\right)}^{n}}\leq C_{\rho ,K}\left\{{||\nabla f_{\rho }||}_{H_{K}^{n}}\sqrt{\lambda }+\frac{s}{\lambda }\right\}, \end{array}$$

where constant *C*_*ρ*,*K*_ relies on *ρ* and *K*. Theorem 10 and these estimates reveal that with confidence 1 − *δ*, we yield

(62) f→tz−∇fρ(LρV2)n≤C~'1+∇fρHKn ×log⁡4δt2γmΠ/∑i=1kmΠisn+2+t2γ+α−1+stγ+t−γ/2.

The learning rate (58) is determined according to the selection of the parameters.

## 5. Experiments

To show the effectiveness of our new ontology algorithms, two experiments concerning ontology measure and ontology mapping are designed below.

### 5.1. Ontology Similarity Measure Experiment on Plant Data

**Figure 1:**
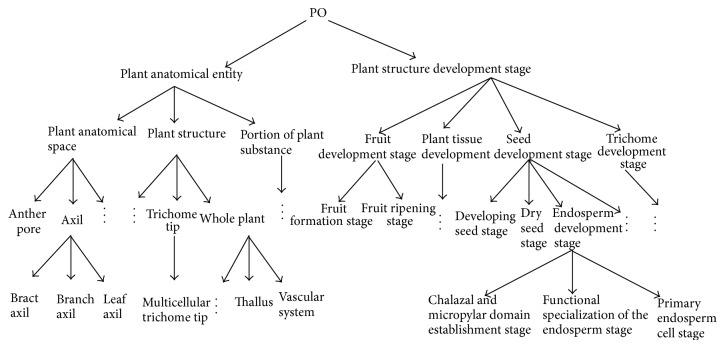
The structure of “PO” ontology.

In the first experiment, we use plant “PO” ontology *O*_1_ which was constructed in the website http://www.plantontology.org/. The structure of *O*_1_ is presented in Figure 1. *P*@*N* (precision ratio; see Craswell and Hawking [24]) is used to measure the quality of the experiment data. Here, we take *k* = 2, *t* = 3, *η*_*t*_ = 1, and *λ* = 0.1.

We first give the closest *N* concepts for every vertex on the ontology graph by experts in plant field, and then we obtain the first *N* concepts for every vertex on ontology graph by Algorithm 3 and compute the precision ratio. Specifically, for vertex *v* and given integer *N* > 0. Let Sim_*v*_^*N*,expert^ be the set of vertices determined by experts and it contains *N* vertices having the most similarity of *v*. Let

(63)  vv1=arg min⁡v'∈V(G)−vfv−fv', vv2=arg min⁡v'∈V(G)−{v,vv1}{fv−fv'},  ⋮ vvN=arg min⁡v'∈V(G)−{v,vv1,…,vvN−1}{fv−fv'}, SimvN,algorithm={vv1,vv2,…,vvN}.

Then the precision ratio for vertex *v* is denoted by

(64) $$\begin{array}{c} {\text{Pre}}_{v}^{N}=\frac{\left|{\text{Sim}}_{v}^{N,\text{algorithm}}\cap {\text{Sim}}_{v}^{N,\text{expert}}\right|}{N}. \end{array}$$

The *P*@*N* average precision ratio for ontology graph *G* is then stated as

(65) $$\begin{array}{c} {\text{Pre}}_{G}^{N}=\frac{\sum_{v\in V(G)}^{}{\text{Pre}}_{v}^{N}}{\left|V\left(G\right)\right|}. \end{array}$$

At the same time, we apply ontology methods in [11–13] to the “PO” ontology. Calculating the average precision ratio by these three algorithms and comparing the results to Algorithm 3 rose in our paper, part of the data is referred to in Table 1.

When *N* = 3, 5, or 10, the precision ratio by virtue of our gradient computation based algorithm is higher than the precision ratio determined by algorithms proposed in [11–13]. In particular, when *N* increases, such precision ratios are increasing apparently. Therefore, the gradient learning based ontology Algorithm 3 described in our paper is superior to the method proposed by [11–13].

### 5.2. Ontology Mapping Experiment on Humanoid Robotics Data

**Figure 2:**
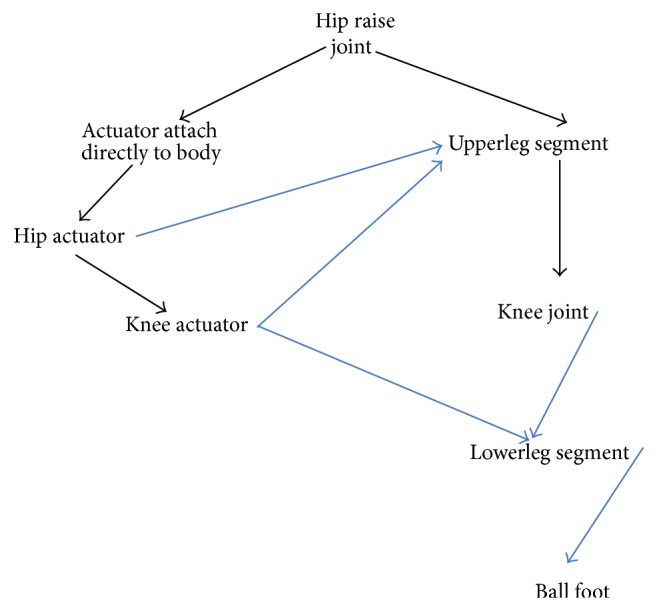
“Humanoid robotics” ontology *O*_2_.

**Figure 3:**
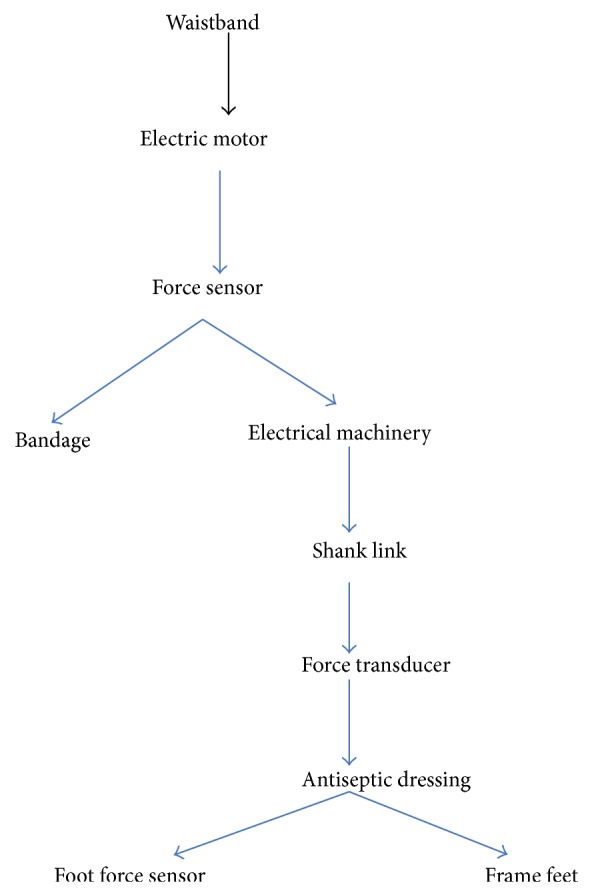
“Humanoid robotics” ontology *O*_3_.

For the second experiment, we use “humanoid robotics” ontologies *O*_2_ and *O*_3_. The structure of *O*_2_ and *O*_3_ is shown in Figures 2 and 3, respectively. The ontology *O*_2_ presents the leg joint structure of bionic walking device for six-legged robot, while the ontology *O*_3_ presents the exoskeleton frame of a robot with wearable and power-assisted lower extremities. In this experiment, we take *k* = 2, *t* = 4, *η*_*t*_ = 1, and *λ* = 0.05.

The goal of this experiment is to give ontology mapping between *O*_2_ and *O*_3_. We also use *P*@*N* precision ratio to measure the quality of experiment. Again, we apply ontology algorithms in [12, 13, 17] on “humanoid robotics” ontology and compare the precision ratio which is gotten from three methods. Some results referred to in Table 2.

Taking *N* = 1, 3, or 5, the precision ratio in terms of our gradient computation based ontology mapping algorithm is higher than the precision ratio determined by algorithms proposed in [12, 13, 17]. Particularly, as *N* increases, the precision ratios in view of our algorithm are increasing apparently. Therefore, the gradient learning based ontology Algorithm 4 described in our paper is superior to the method proposed by [12, 13, 17].

## 6. Conclusions

As a data structural representation and storage model, ontology has been widely used in various fields and proved to have a high efficiency. The core of ontology algorithm is to get the similarity measure between vertices on ontology graph. One learning trick is mapping each vertex to a real number, and the similarity is judged by the difference between the real number which the vertices correspond to. In this paper, we raise a gradient learning model for ontology application in multidividing setting. The sample error and approximation properties are given in our paper. These results support the gradient computation based ontology algorithm from the theoretical point of view. The new technology contributes to the state of the art for applications and the result achieved in our paper illustrates the promising application prospects for multidividing ontology algorithm.

## Figures and Tables

**Table 1:** The experiment results of ontology similarity measure.

	*P*@3 average precision ratio	*P*@5 average precision ratio	*P*@10 average precision ratio
Algorithm 3 in our paper	0.5042	0.6216	0.7853
Algorithm in [11]	0.4549	0.5117	0.5859
Algorithm in [12]	0.4282	0.4849	0.5632
Algorithm in [13]	0.4831	0.5635	0.6871

**Table 2:** The experiment results of ontology mapping.

	*P*@1 average precision ratio	*P*@3 average precision ratio	*P*@5 average precision ratio
Algorithm 4 in our paper	0.4444	0.5185	0.6111
Algorithm in [17]	0.2778	0.4815	0.5444
Algorithm in [12]	0.2222	0.4074	0.4889
Algorithm in [13]	0.2778	0.4630	0.5333
